# Pregnancy without Emissio-Penis

**Published:** 1864-07

**Authors:** 


Pregnancy Without Emissio-Penis.—Prof. Scanzoni relates the case of a woman, aged 29, in whom he detected a four months' pregnancy, although the orifice of the vagina was closed bp a firm and tense membrane, in which an aperture big enough to admit a probe was discovered with much difficulty. In his long experience he had never met with a similar instance, although he has seen cases in which remains of the hymen exsted, and one in which this membrane continued quite uninjured. The membrane only yielded slightly uwards, so that emissio-penis was completely impossible, and the question is, how did the semen reach the uterine orifice four inches distant? After the seventh month the opening gradually widened a little, so that a quill could be easily introduced; and by the time labour set in the finger could be passed in. As the labour advanced, there was found to be also a thin circular membrane attached to the walls of the vagina at the junction of its upper and middle third; but a large opening existed in this, so that it caused no obstruction to delivery. As, however, the thickened hymen formed a dense ring, which prevented the passage of the head, a small crucial incision was performed, and the delivery easily terminated.—*Alleg. Wien Med. Zeit.*, No. 4. [Dr. Mattei, *Union Medicale* No. 36, relates a similar case of a woman who became pregnant after having been married 11 years. The husband was aware that she was malformed, but had contented himself with incomplete connection. On examination, a cul-de-sac was found, which, probably formed through the attempts at copulation, only admitted the finger to the extent of 1½ centimetre. The most careful examination by means of the speculum, could not detect the aperture by which the semen must have entered. After severe labor for three days, the tissues in front of the head gave way and admitted the finger. Delivery was completed by the forceps].—*Medical Times and Gaz.*, May 28, 1864.—*Med. News.*

